# P-1660. Clinical Microbiology of Xylazine-Associated Wound Infections

**DOI:** 10.1093/ofid/ofae631.1826

**Published:** 2025-01-29

**Authors:** Deanna Berg, Christina Maguire, Amanda Binkley, Jessie Torgersen, Matthew Hinton, Derek Peiffer, Samantha Huo, Sean Foster

**Affiliations:** Penn Presbyterian Medical Center, Philadelphia, PA; Penn Presbyterian Medical Center, Philadelphia, PA; Penn Presbyterian Medical Center, Philadelphia, PA; Penn Presbyterian Medical Center, Philadelphia, PA; Penn Presbyterian Medical Center, Philadelphia, PA; Penn Presbyterian Medical Center, Philadelphia, PA; University of Pennsylvania Health System, Philadelphia, Pennsylvania; Penn Presbyterian Medical Center, Philadelphia, PA

## Abstract

**Background:**

Over 90% of illicit opioids in Philadelphia contain xylazine, a potent centrally acting α-2 adrenergic agonist that can promote the development of skin and soft tissue wounds with a subsequent risk of infection. Optimal wound management, including the need for broad antimicrobial coverage, remains uncertain. This study sought to describe culture positivity and the clinical microbiology of xylazine-associated wounds to inform the optimal antimicrobial spectrum of activity.

**Figure:**
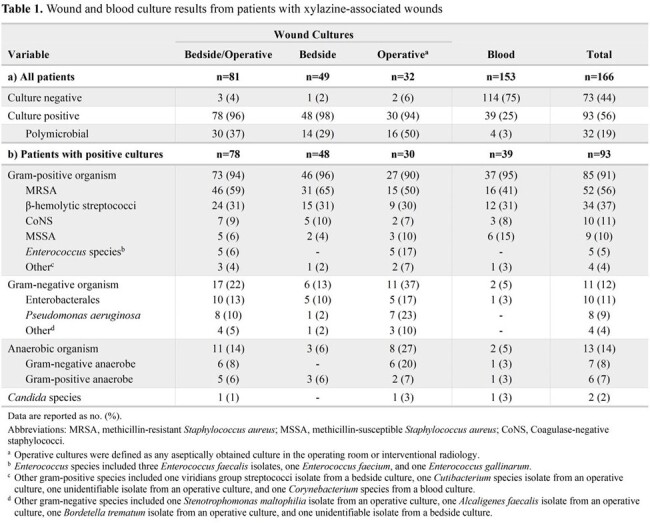

**Methods:**

We conducted a retrospective, single-center, cross-sectional study of adults with xylazine-associated wounds between April 1, 2022, and December 1, 2023. Patients were included if they received antimicrobials for suspected wound infection related to injection drug use, along with one of the following: (1) a positive urine xylazine test, (2) a positive urine fentanyl test with patient-reported xylazine use. For patients with multiple hospitalizations and/or wound cultures, the earliest encounter with the single most invasive method of wound culture acquisition was utilized. Microbiology results and causative pathogens are reported.

**Results:**

Of the 685 patients with positive urine toxicology, 166 were included in the analysis, and 56% had positive cultures. Wound cultures were obtained in 81 (49%) patients; superficial bedside cultures were the only culture type obtained in 61% of patients, and operative cultures were collected in 39% of patients. Methicillin-resistant *Staphylococcus aureus* (MRSA) was the most common causative pathogen, and *Pseudomonas aeruginosa* was isolated in 9% of patients, most of whom had a bone or joint infection (88%). Nearly one in four patients were bacteremic (Table 1).

**Conclusion:**

Over half of patients with suspected infected xylazine-associated wounds had clinical microbiology results to guide antimicrobial therapy. Gram-positive organisms were most frequently isolated, while gram-negative organisms were infrequently observed. These observations suggest that suspected infected xylazine-associated wounds should include empiric coverage of MRSA and β-hemolytic streptococci. Empiric anti-pseudomonal and/or anaerobic coverage may not be necessary for most patients being treated for skin and soft tissue infections.

**Disclosures:**

**Christina Maguire, PharmD**, Viiv: Advisor/Consultant **Amanda Binkley, PharmD, BCIDP, AAHIVP**, Viiv: Advisor/Consultant

